# Voltammetric Electronic Tongues in Food Analysis

**DOI:** 10.3390/s19194261

**Published:** 2019-09-30

**Authors:** Clara Pérez-Ràfols, Núria Serrano, Cristina Ariño, Miquel Esteban, José Manuel Díaz-Cruz

**Affiliations:** 1 Department of Chemical Engineering and Analytical Chemistry, University of Barcelona, Martí i Franquès 1-11, E08028 Barcelona, Spain; claraperezrafols@ub.edu (C.P.-R.); nuria.serrano@ub.edu (N.S.); cristina.arino@ub.edu (C.A.); miquelestebanc@ub.edu (M.E.); 2 Institut de Recerca de l’Aigua (IdRA) of the University of Barcelona. Martí i Franquès 1-11, E08028 Barcelona, Spain

**Keywords:** voltammetric electronic tongues, food analysis, food authentication

## Abstract

A critical revision is made on recent applications of voltammetric electronic tongues in the field of food analysis. Relevant works are discussed dealing with the discrimination of food samples of different type, origin, age and quality and with the prediction of the concentration of key substances and significant indexes related to food quality.

## 1. Introduction

**Figure 1:**
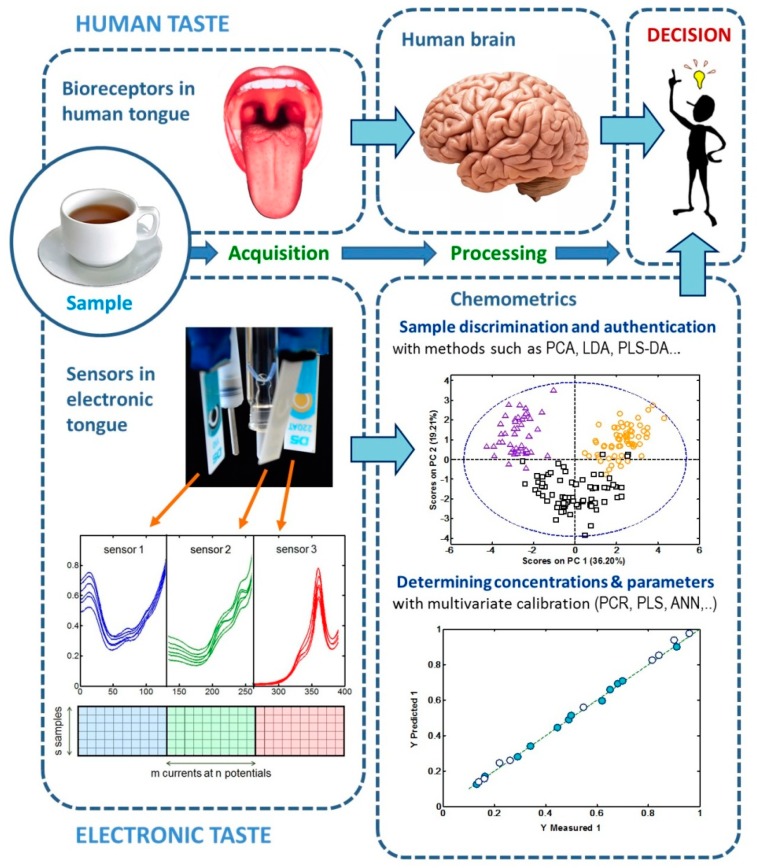
General scheme of voltammetric electronic tongues.

Electronic noses and electronic tongues are bioinspired devices created during the 1980’s as a successful encounter of electronics and electrochemistry with chemometrics. As suggested by their names, they try to mimic the ability of human noses and human tongues to identify characteristic odors and tastes [1,2,3,4,5,6]. For this purpose, an array of non-specific sensors is used to mimic the role of the diverse bio-receptors present in the human nose/tongue and a chemometric model is used to mimic the processing of the bio-receptor’s signals by the human brain, as Figure 1 shows. Regarding the sensors, they must present different sensitivities (cross response) with respect to the substances related to the odor/taste, so that these sensors are able to provide complementary, non-redundant information. As for chemometric models, they can be used to classify the samples (e.g., according to different protected designations of origin, PDO) or to quantitatively determine the concentrations of target species (e.g., major components, adulterants, pollutants) or the values of parameters related to some food properties (e.g., antioxidant capacity, bitterness).

Although electronic noses and tongues are employed in many research fields such as environmental monitoring [7], pharmacy [8] or biotechnology [9], these devices have been mostly focused on food analysis [10,11,12,13]. This is not strange, since they try to mimic human noses and tongues, which are frequently busy smelling and tasting food products. As a consequence, a large number of works have been published in recent years dealing about the application of electronic noses and tongues to food analysis, as the reviews in [10,11,12,13] show.

In this work we will focus on voltammetric electronic tongues, proposed by Winquist et al. in 1997 [14] as an alternative/complement to the existing potentiometric electronic tongues, mostly based on measurements with ion-selective electrodes and frequently applied in food analysis [12]. Figure 1 shows an example of voltammetric electronic tongue constituted by three screen-printed devices acting as sensing units. Although every screen-printed unit includes all electrodes needed for a voltammetric measurement (working, reference and auxiliary), the tongue shown in the picture also has conventional reference and auxiliary electrodes for a more accurate control of the potential applied. Then, a multichannel potentiostat simultaneously registers the current of the three working electrodes of the screen-printed devices as a function of the potential. The voltammograms measured by all three sensors (depicted in blue, green and red) are integrated into a data matrix (where the position of the datasets coming from the different sensors is indicated with the same colors). Then, the matrix is submitted to a chemometric strategy to get qualitative and/or quantitative information.

In the first case, a pattern recognition tool like principal component analysis (PCA) can be applied to detect groups of samples with common properties (clusters). In the PCA scores plot shown in the figure above, three clusters are visible, which agree very well with the previous information about the origin of the samples (denoted with purple, yellow and black colors). If the PCA model can identify the origin of known samples (training set), then it can be used to identify unknown samples. In the case of quantitative information, a calibration model is built with a method like partial least squares (PLS) applied to a training set of samples with known values of the properties to be determined. Then, if the predictions of the calibration model are right (as shown by the predicted versus measured plot in Figure 1) it can be used to predict the desired properties in unknown samples from the voltammograms acquired by the tongue.

As compared to potentiometric devices, the advantages of voltammetric electronic tongues include the higher amount of information achieved (a full voltammogram instead of a single potential per sensor) and the higher sensitivity (especially when electrochemical preconcentration is possible), which allows one to consider not only major components of the sample but also trace substances that can be very informative about the origin and quality of food products. However, the main drawbacks of voltammetric devices are the higher complexity of the experimental design and, especially, of the datasets, which usually require sophisticated chemometric models for the data treatment. Thus, although it is clear that potentiometric tongues are a simple, compact and user-friendly solution in many cases, voltammetric tongues can be a more powerful solution for complicated problems. The real power of the ‘voltammetric way’ is conditioned, on the one hand, by the sensitivity and (cross) selectivity of the sensors integrating the array and, on the other hand, by the performance of the chemometric method used for the data treatment.

Concerning the sensors integrating the array, two main trends can be identified. Some research groups prefer arrays of bare metals such as silver, gold, platinum, or iridium combined with high and low amplitude pulsed signals at different frequencies [15], whereas other groups select electrode substrates chemically modified with substances that present certain affinity for the target species combined with the potential scans typically used in voltammetry (e.g., linear sweep, differential pulse, square wave). In the last case, modifications can be made on many substrates such as carbon paste [16], graphite–epoxy composites [17], glassy carbon [18] or screen-printed electrodes [19].

As for the chemometric tools, methods like PCA, linear discriminant analysis (LDA) or partial least squares discriminant analysis (PLS-DA) are applied for sample discrimination, whereas multivariate calibration methods like principal component regression (PCR) or PLS are used for determining concentrations and quality parameters in the case of reasonably linear data [20,21,22,23,24]. When measurements behave in a strongly non-linear way, more sophisticated methods like artificial neural networks (ANN) or support vector machine (SVM) are employed [23,24,25,26]. Figure 1 summarizes how both sensing and data treatment strategies applied to voltammetric electronic tongues converge to mimic human tongues.

The main body of this review is divided into two parts, a first one dedicated to the characterization, classification and authentication of food products and a second part regarding the determination of chemical species and other quantitative parameters. For more general information about the use of voltammetric electronic tongues in food analysis we refer to the more extensive review by Wei et al. [27].

## 2. Characterization, Classification and Authentication of Food Products

As already pointed out, voltammetric electronic tongues can provide valuable qualitative information about food samples when combined with a chemometric method of pattern recognition. Table 1 summarizes some relevant applications found in references [14,15,16,17] and [28,29,30,31,32,33,34,35,36,37,38,39,40,41,42,43,44,45,46,47,48,49,50,51,52,53,54,55,56,57,58,59,60,61,62,63,64,65,66,67,68,69,70,71] focused on the characterization, classification and authentication of food products.

In general terms, voltammetric tongues are mostly applied to liquid samples, with especial predominance of wine, but they can also operate with more consistent food products such as honey, yogurt, meat or fish. The number of working electrodes ranges from two to eight and includes bare metals, carbon paste electrodes modified with phthalocyanine and other substances, graphite–epoxy based electrodes and screen-printed electrodes. As already mentioned, arrays of bare metals usually are submitted to multi-pulse excitation signals whereas the rest of the sensors mostly work with cyclic (CV), square wave (SW) or differential pulse voltammetric (DPV) scans. In some cases (especially CV), the scans generate a large amount of data and some compression is needed to increase the speed of calculations. The simplest solution is to replace the full voltammogram by a few parameters describing relevant features. For instance, Bougrini et al. [60] used the difference between the maximum and minimum values of the current and the maximum slope of the current curve in the anodic and cathodic scans. In this way, the measurements of a honey sample with an array of seven working electrodes produces just a set of 21 numbers. The PCA treatment of these roughly compressed data allowed a quite satisfactory discrimination among 13 types of honey. However, in some cases this method of compression can lead to the loss of valuable information and it is better to resort to more sophisticated compression strategies like fast Fourier transform (FFT) or discrete wavelet transform (DWT) which preserve most of the information of the voltammogram with a significant decrease in the amount of data. For instance, Cetó et al. [37] obtained good results with the DWT compression of CV data from five sensors in the PCA discrimination of different cava wine varieties (results that were further refined by means of ANN).

In many situations, a relatively simple and unsupervised data treatment by means of PCA is sufficient to visually discriminate groups of samples in the scores plot. However, more complex problems require supervised classification methods like LDA or PLS-DA where the classes of the known samples are included in a model that will be used to assign unknown samples to the predefined classes. This is the case, for instance, of the work by Blanco et al. [48] that will be further discussed.

As for the discrimination purposes, most studies are focused either on the authentication of both local origin (typically PDO) and quality of food products or on the detection of adulterations. Nevertheless, there is an increasing interest on the evolution of products with time (e.g., grape ripening, spoilage of meat and fish during storage) in order to detect the samples that are not in good conditions to be consumed. Especially interesting is the work by Haddi et al. [70], which used measurements by CV in a tongue of seven working electrodes to discriminate the type of meat (beef, goat or sheep) and the degree of spoilage, measured in terms of number of storage days.

The same data sets used for sample discrimination can be processed by multivariate calibration methods like PLS to predict certain parameters related to food quality. For this purpose, it is necessary to measure the target parameter in the known samples and include the resulting values in the calibration model. Anyway, these quantitative implications of electronic tongues will be discussed in Section 3.

**Figure 2:**
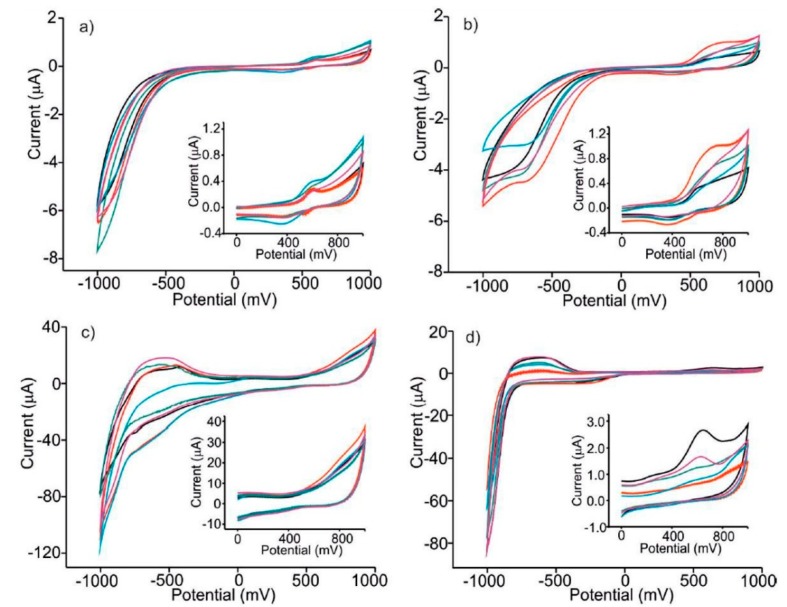
Cyclic voltammograms registered using four different carbon paste electrodes (CPEs) immersed in liqueur samples made from different varieties of apples. (**a**) Unmodified CPE; (**b**) ZnPc–CPE; (**c**) FePc–CPE; (**d**) CoPc–CPE. Apple varieties: Ligol (black), Kosztela (red), Grey Reinette (blue), Rubin (green), Cox Orange (purple). Reproduced from [43].

As a first example of sample discrimination with voltammetric electronic tongues, we will briefly discuss the work by M. Sliwinska et al., published in [43] and focused on apple liqueurs (Nalewka). In this study, an array of four working electrodes was used, including an unmodified carbon paste electrode (C-CPE) and three electrodes chemically modified with cobalt, iron and zinc phthalocyanines (denoted as CoPc–CPE, FePc–CPE and ZnPc–CPE, respectively). Figure 2 compares the cyclic voltammograms registered for liqueurs prepared with five different apple varieties (Ligol, Kosztela, Grey Reinette, Rubin and Cox Orange). Notorious differences can be observed between the responses obtained with different electrodes and in different types of liqueur.

**Figure 3:**
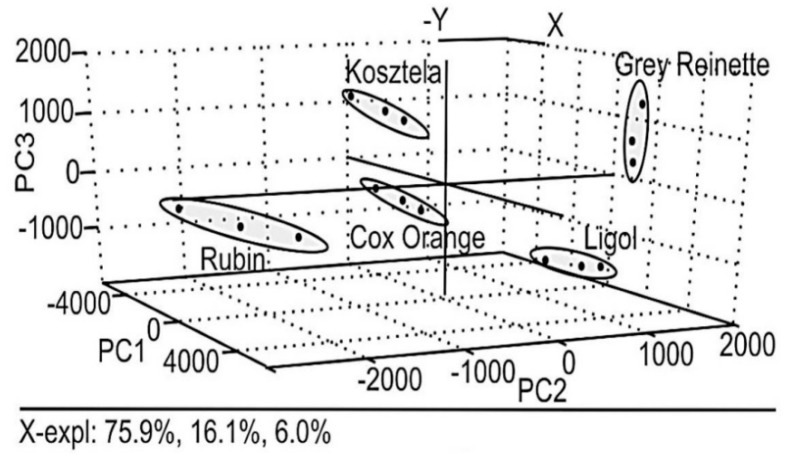
Scores plot obtained in the PCA treatment of cyclic voltammetric (CV) data (see Figure 2) acquired with four working electrodes in three replicates of five types of apple liqueur (from Ligol, Kosztela, Grey Reinette, Rubin and Cox Orange apples). Reproduced from [43].

The application of PCA to this kind of data allowed the authors to clearly discriminate among liqueurs made from different varieties of apple, as shown by Figure 3. This is a 3D plot of the scores achieved by the three samples of every apple variety for the three first principal components of the model (PC1, PC2 and PC3). As it can be seen, the three replicates of each variety are very close with each other in the graph and quite far from the groups of replicates of the other varieties, which makes it possible to identify unknown samples from the position of their scores in the diagram.

**Figure 4:**
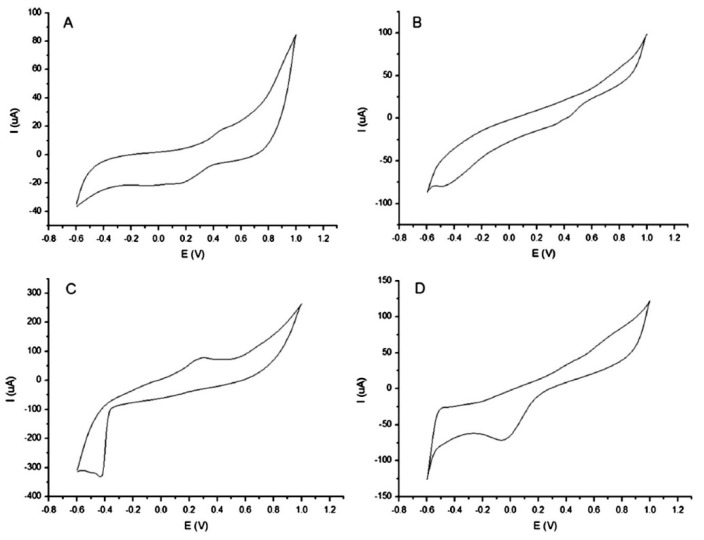
Cyclic voltammograms registered with an array of four commercial screen-printed sensors exposed to a beer sample. (**A**) DS-110; (**B**) DS-250AT; (**C**) DS-410; and (**D**) DS-550. Reproduced from [48] with permission.

**Figure 5:**
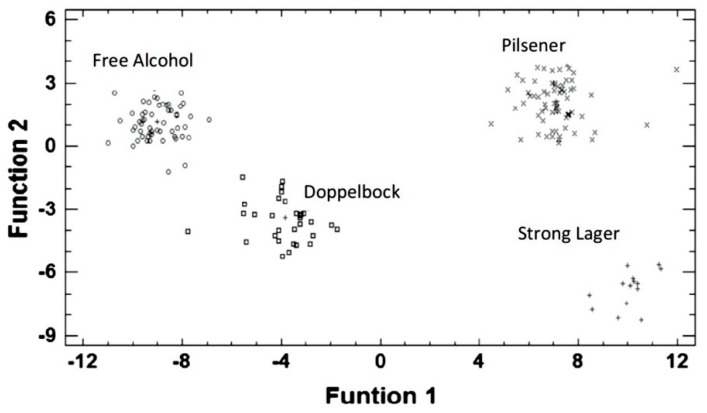
Scores plot obtained in the LDA treatment of CV data (see Figure 4) acquired with four commercial screen-printed sensors in beer belonging to three different categories. Reproduced from [48] with permission.

As pointed out before, supervised classification methods like LDA and PLS-DA can improve the performance of PCA in more intricate situations, as it happens in our second example. Blanco et al. [48] applied LDA to CV data from four commercial screen-printed electrodes (Figure 4) to classify beer samples in four categories: free alcohol, Pilsener, doppelbock and European strong lager. In LDA, new optimized variables (discrimination functions, DFs) are obtained from the original variables but, unlike the principal components (PCs) used in PCA, such variables do not try to explain most of the data variance. They are constructed to get the maximum discrimination between the predefined classes. Then, a plot of the scores of the discriminant functions (Figure 5) allows a satisfactory classification of the beers which was not possible with PCA (data not shown).

## 3. Determination of Chemical Species and Other Quantitative Parameters Related to Food Analysis

Unlike human tongues, voltammetric electronic tongues can be used for quantitative purposes if their signals are submitted to a multivariate calibration method such as PLS or ANN. For instance, they can be applied to determine pollutants like nitrophenols [72], heavy metal ions [73] or glyphosate [74] in different samples. In the case of food analysis, predictions can be made not only of the concentrations of relevant species, but also of a large deal of parameters informing about the quality of food products. Table 2 summarizes some representative contributions in this field, corresponding to references [75,76,77,78,79,80,81,82,83,84,85,86,87,88,89,90]. It can be seen that the working electrodes used are essentially the same employed for sample discrimination (Table 1). Among the substances determined we can mention theaflavine and thearubigin in tea, several sugars in fruits and sugar cane bagasse, bisulfite and ethylphenol metabolites in wine, some anions in meat and antibiotic residues in milk. As for the quality parameters, the determination of bitterness, polyphenol indexes and antioxidant capacity in wines, beers and olive oils deserves special attention. Concerning chemometric tools, PLS (and, eventually, PCR) is the main choice, but non-linearity problems quite often demand more sophisticated methods like ANN or SVM.

**Figure 6:**
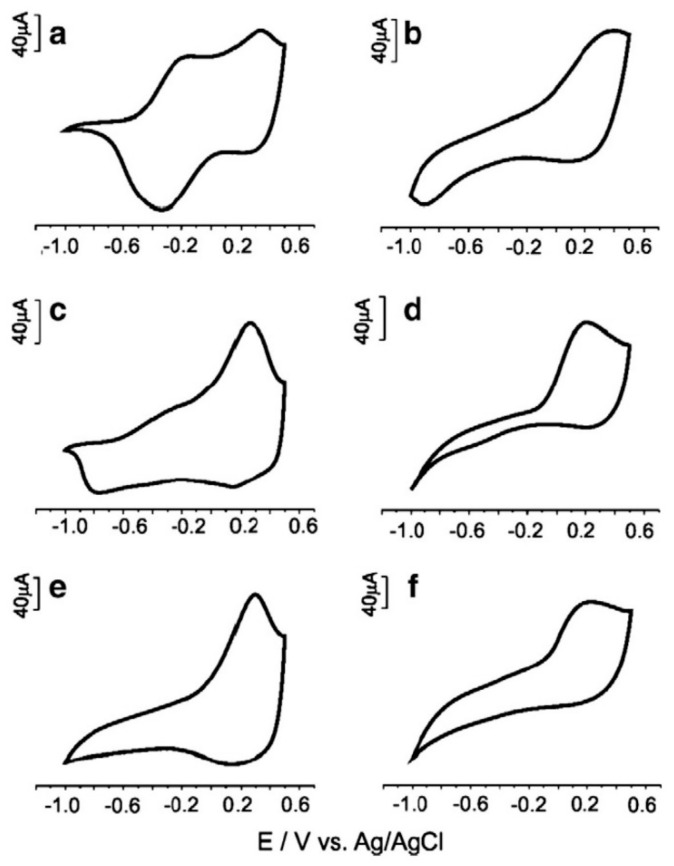
Cyclic voltammograms measured with the six working electrodes described in [65] in the same emulsion of extra virgin olive oil. (**a**) Ppy/FCN; (**b**) Ppy/MO; (**c**) Ppy/NP; (**d**) Ppy/AQS; (**e**) Ppy/H2SO4; (**f**) Ppy/PWA Reproduced with permission.

**Figure 7:**
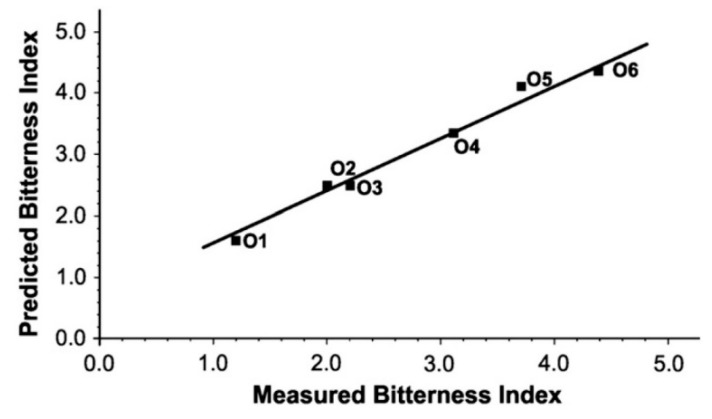
Plot of the bitterness index predicted with the electronic tongue in [65] by means of PLS calibration for different olive oil samples as a function of the corresponding bitterness index obtained by a chemical method. Reproduced with permission.

As an example of these studies, we will summarize the work by Apetrei, who in [65] not only classified different extra virgin olive oils according to their degree of bitterness, but also applied a multivariate calibration model to predict the corresponding bitterness indexes. For this purpose, six polypyrrole-based screen-printed electrodes were used to measure quite different cyclic voltammograms as these shown in Figure 6. Then, a PLS model was constructed with the voltammograms of calibration samples whose bitterness indexes had been previously determined by means of a chemical method. PLS is amongst the most popular multivariate calibration methods and is present in commercial software. It is based on a calibration between the experimental data matrix and that containing the parameters to be predicted, where both matrices have been ‘compressed’ in terms of a set of optimized variables called latent variables (LVs). The main difference of LVs with the PCs of PCA is that they are optimized to maximize the covariance between both matrices involved in the calibration. Although PLS processing provides many useful graphs, most authors just provide the errors of the calibration (RMSEC) and the validation (RMSEV) in a table. Fortunately, a few authors like Apetrei also provide the plot of the predicted values as a function of the real ones (Figure 7). In a good PLS model the points of this graph should be placed in a straight line, not very far from the theoretical line of slope 1 and intercept 0. As Figure 7 shows, the PLS model in [65] is quite successful in predicting bitterness indexes.

**Figure 8:**
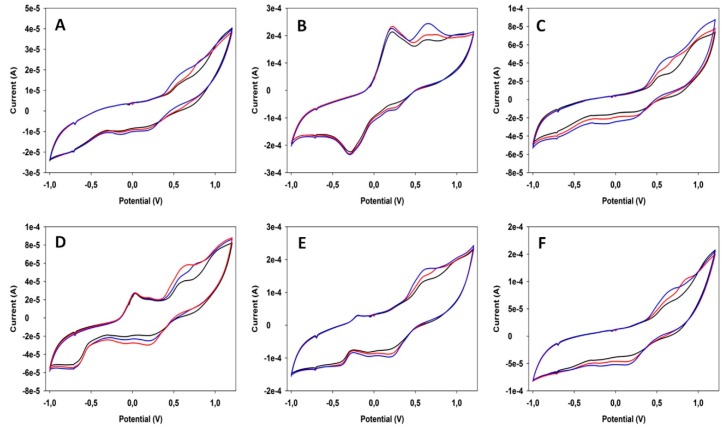
CV signals obtained for 25 ppm of 4-EP (black), 4-EG (red) and 4-EC (blue) in a wine matrix for (**A**) a bare epoxy-graphite electrode; and electrodes modified with (**B**) Cu nanoparticles; (**C**) WO_3_ nanoparticles; (**D**) Bi_2_O_3_ nanoparticles; (**E**) polypyrrole; and (**F**) Co(II) phthalocyanine. Reproduced from [85] with permission.

**Figure 9:**
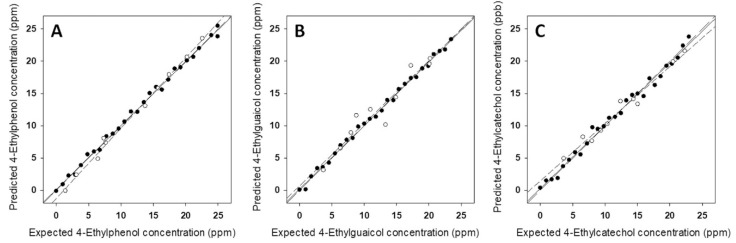
Modeling ability of the developed ANN: expected vs. predicted concentrations for (**A**) 4-EP; (**B**) 4-EG; and (**C**) 4-EC, both for training (•, solid line) and testing subsets (○, dashed line). Dotted line corresponds to theoretical diagonal line. Reproduced from [85] with permission.

In general terms, PLS method works well for reasonably linear data. However, for strongly non-linear data PLS fails and more sophisticated data treatments are required, like ANN or SVM. In our last example, taken from ref. [85], González-Calabuig and del Valle used ANN to predict the content in wine samples of the metabolites related to the Brett defect: 4-ethylphenol (4-EP), 4-ethylguaiacol (4-EG) and 4-ethylcatechol (4-EC). For this purpose, they registered CV scans in wine samples spiked with different concentrations of metabolites by using a voltammetric electronic tongue integrated by six bulk-modified graphite–epoxy composites and obtained signals like those shown in Figure 8. The original data (2490 inputs per sample) were compressed by means of DWT with a 93.5% compression ratio (132 numbers per sample) to keep the maximum information with the minimum data size. Then, an ANN model was built with 27 standards containing different proportions of the three analytes (according to a 3^3^ factorial design). The model is composed by neurons, i.e., calculation units organized in layers (usually three) which use transfer functions that operate the numbers in the preceding layer to generate the numbers in the next layer. A critical point in ANN strategy is to design the architecture of the network (e.g., the neurons per layer or the type of transfer functions). In this case, a network was built with 132 neurons in the input layer, 3 neurons in the hidden layer and 3 neurons in the output layer. The training of the ANN consists on the optimization of the coefficients of the transfer functions to get results in the output layer (the concentrations of the three analytes) as close as possible to the real ones when the compressed measurement of the 27 standards are fed to the input layer. As Figure 9 shows, the trained ANN produced good predictions for all three analytes not only in the standard solutions (denoted with black circles), but also in 10 additional solutions used for external validation (empty circles).

## 4. Conclusions

In the field of food control, voltammetric electronic tongues are a promising complement for the more widely used potentiometric electronic tongues and electronic noses, especially in these really complex situations demanding a higher amount of information. For data sets not too far from linearity, conventional chemometric methods such as PCA, LDA, PLS or PLS-DA can be applied to build models that are able to discriminate between samples of different origin and freshness or to determine the concentration of key substances and relevant indexes related to food quality. In the presence of strongly non-linear data, more sophisticated chemometric methods such as ANN or SVM can be applied instead. The problem of these methods, however, is that their use is not obvious. For instance, ANNs require a careful design of their architecture, which usually is made by means of the trial and error strategy. As a consequence, models are built for particular situations and datasets, and are rarely accessible as supplementary materials of the papers or in the webs of research groups. Then, they promote a sensation of ‘black box’ that, unlike PCA or PLS, prevents their dissemination as a common practice in the treatment of voltammetric tongue data.

Nowadays there is a great diversity of designs and applications of voltammetric electronic tongues, recently enhanced by the popularization of screen-printed electrodes. In our view, the initial strategy of pulse activation signals and metallic electrodes has been progressively replaced by conventional voltammetric scans (mainly CV) and chemically modified electrodes which enhance the (cross) selectivity of the sensors constituting the tongue. We believe that this is a positive trend, since chemical interactions of significant components of the sample with the modified electrodes are more likely to produce sample discrimination than just the different electrochemical behavior of electrodes made of different metals. Nevertheless, in our opinion, an effort is required to achieve the maximum simplicity and economy in both the electrode selection and the strategy for data treatment. This means that statistic tools should be applied to confirm that all the sensors used are really necessary (eight sensors for a tongue predicting a single parameter sounds a bit excessive). Additionally, powerful compression methods like DWT or FFT should be extensively applied to the data sets to reduce the computing time (in their competition with chromatographic methods, voltammetric tongues should not spend in calculations the time saved for the absence of separation). Finally, an effort should be made to adapt calibration models to matrix effects and signal drifts in order to improve their reliability in real operating conditions. For this purpose, multivariate adaptations of the univariate methodologies of standard addition and inner standard would be highly welcome.

Just to conclude, only with robust and reproducible electrode arrays susceptible to cost-effective mass production and with programs for data treatment implemented in commercial software voltammetric electronic tongues will be able to cross the frontier between the proof of concept and the market for an effective assessment of food provenance and quality.

## Figures and Tables

**Table 1:** Selected works dealing about the characterization, classification and authentication of food products with voltammetric electronic tongues.

Food Product	Working Electrodes	Data Analysis	Comments	Ref.
Orange juice and milk	Pt and Au	PCA	First voltammetric e-tongue	[14]
Milk	Au, Pt, Rh, stainless steel	PCA	Monitoring of milk in dairy industry	[28]
	Au, Cu, Au modified with Prussian blue	PCA	Recognition of milk adulteration with hydrogen peroxide	[29]
	Au, Cu, Pt	PCA	Recognition of milk adulteration with urea, formaldehyde and melamine	[30]
	Au, Pd, Pt	MPCA, NPLS-DA	Recognition of milk adulteration with urea	[31]
	Au, Pt, Ag	PCA, PLS-DA	Discrimination of various brands of pure milk	[32]
	Au, Ag, Pt, Pd	PCA, CA	Monitoring of quality and storage time of unsealed pasteurized milk	[15]
Yogurt	Au, Ag, Pt, Pd	SVM	Monitoring the fermentation, post-ripeness and storage processes of set yogurts	[33]
	Au, Ag, Pt, Pd	PCA, CA	Evaluation of varieties of set yogurts	[34]
Wines and liqueurs	Phthalocyanine-based carbon paste electrodes and electrodes covered with conducting polypyrrole doped with different counter ions	PCA	Detection of adulterations in wines	[16]
	Au, Cu	PCA	Discrimination of wines and whiskies of different quality	[35]
	Au, Ag, Pt, Pd, W, Ti	PCA, CA	Classification of rice wines of different ages	[36]
	Five bulk-modified graphite-epoxy electrodes	PCA, ANN	Cava wine authentication	[37]
	Six bulk-modified graphite-epoxy electrodes	LDA	Cava wine authentication	[17]
	Five different graphite-epoxy composite electrodes	PCA, ANN	Discrimination of wines of different types and PDO	[38]
	Sensors based on metallic and bulk-modified graphite electrodes	LDA	Classification of wines of different PDO	[39]
	Four carbon paste electrodes chemically modified in different ways	PCA	Discrimination between red wines aged in oak barrels and matured in steel tanks in contact with oak wood chips	[40]
	Three nanocomposites modified electrodes prepared with Au and Cu nanoparticles in the presence of conducting polymers and carbon nanotubes.	PCA, LDA	Classification of rice wines of different geographical origins	[41]
	Six modified epoxy-composite electrodes	LDA	Classification of brandies according to their taste category and ageing method	[42]
	Four carbon paste electrodes: one unmodified and the others chemically modified with Co, Fe and Zn phthalocyanines	PCA	Discrimination of apple liqueurs	[43]
Grapes	Eight metallic electrodes housed inside a stainless steel cylinder	PCA	Study of grape ripening	[44]
	Poly-ethylendioxythiophene modified Pt electrode and sonogel carbon electrode	PCA	Study of grape ripening	[45]
Beer	Three enzymatic biosensors based on tyrosinase and phthalocyanines as mediators	PCA, LDA	Monitoring of the aging of beers	[46]
	Six bulk-modified graphite-epoxy electrodes	PCA, LDA, PLS-DA	Classification of three types of beer: Lager, Stout and IPA	[47]
	Four commercial screen-printed electrodes made of carbon, Au, carbon/Co-Phtahlocyanine and Pt	PCA, LDA	Classification of different types of beer	[48]
Coffee	Six graphite-epoxy electrodes modified in different ways	LDA, SVM	Geographical classification of Mexican coffees	[49]
	Au wire and graphite rod	PCA	Discrimination of civet coffee	[50]
Tea	Ir, Pt, Rh	PCA	Discrimination of nine different teas	[51]
	Au, Ir, Pd, Pt, Rh	PCA, LDA	Tea quality assessment	[52,53]
	Pt and glassy C	PCA, LDA	Classification of black tea liquor	[54]
	Metallic oxide-modified nickel foam electrodes (SnO_2_, ZnO, TiO_2_, Bi_2_O_3_)	PCA, SVM	Classification of green and black teas	[55]
	Au, Ir, Pd, Pt, Rh	PCA	Monitoring the fermentation process of black tea	[56,57]
Honey	Au, Ag, Pt, Pd, W, Ti	PCA, CA, DFA	Classification of mono-floral honeys	[58]
	Au, Ag, Pt, Pd, W, Ti	PCA, DFA	Tracing floral and geographical origins of honey	[59]
	Pt, Au, glassy C, Ag, Pd, Ni, Cu	PCA, SVM, HCA	Classification of Moroccan and French honeys according to geographical and botanical origins and detection of adulteration	[60,61]
	Au, Ag, Pt	PCA, LDA	Authentication of mono-floral and honeydew Romanian honeys	[62]
	Ir, Rh, Pt, Au	PCA	Monitoring honey adulteration with sugar syrups	[63]
	Au, Ag, Pt, glass electrode	PLS-DA	Monitoring honey adulteration	[64]
Oil	Six electrodes based on polypyrrole	PCA, PLS-DA	Discrimination of extra virgin olive oils according to their degree of bitterness	[65]
	Pt, Au, glassy C, Ag, Ni, Pd, Cu	PCA, DFA, SVM	Detection of adulteration in argan oil	[66]
	Modified carbon paste electrodes	PLS-DA	Detection of virgin olive oil adulteration	[67]
	Cu, glassy C, Au, Ni, Pd, Pt, Ag	PCA, SVM, HCA	Identification of Portuguese olive oils	[68]
Meat and fish	Screen-printed electrodes modified with bisphthalocyanine and polypyrrole	PCA, PLS-DA	Beef freshness monitoring by detection of ammonia and putrescine	[69]
	Pt, Au, Ag, glassy C, Pd, Cu, Ni	PCA	Assessment the origins of red meats and their storage time	[70]
	Ir, Rh, Pt, Au, Ag, Co, Cu, Ni	PCA	Shelf-life assessment of fresh cod in cold storage	[71]

**Table 2:** Selected works dealing with the determination of chemical species and other quantitative parameters in food analysis by using voltammetric electronic tongues.

Application	Working Electrodes	Data Analysis	Ref.
Prediction of bitterness and alcoholic strength in beers	Polypyrrole polymerized onto Pt disks and doped with different modifiers	PLS	[75]
Determination of total polyphenol index in wines	Five graphite-epoxy electrodes modified in different ways	PLS, ANN	[76]
Determination of theaflavin and thearubigin in black tea	Au, Ir, Pd, Pt, Rh	PLS, SVM, ANN	[77,78]
Evaluation of sugar content and firmness of non-climacteric pears	Au, Ag, Pt, Pd, W, Ti	PLS, PCR, SVM	[79]
Evaluation of the antioxidant capacity of red wines	Graphite-epoxy composite electrodes and modified carbon paste electrodes	PLS, ANN	[80]
Evaluation of oxygen exposure levels and polyphenolic content of red wines	Modified carbon paste electrodes based on bisphthalocyanines and perylenes	PLS	[81]
Quantification in rosé cava wines of different indexes related to total phenolic content and other specific phenolic features	Four graphite–epoxy voltammetric (bio)sensors with different modifiers such as tyrosinase, laccase and copper nanoparticles	ANN	[82]
Determination of galactose, glucose, xylose and mannose in sugar cane bagasse	Glassy carbon electrodes modified with multi-walled carbon nanotubes containing metal (Pd, Au, Cu, Ni, Co)	ANN	[18]
Determination of spring water quality parameters	Ir, Rh, Pt, Au	PLS	[83]
Determination of bisulphites in wines	Au, Rh, Pt, stainless steel	PLS	[84]
Determination of ethylphenol metabolites in wines	Six graphite–epoxy modified composite electrodes	ANN	[85]
Determination of nitrate, nitrite and chloride in minced meat	Au, Pt, Rh, Ir, Ag, Ni, Co, Cu	PLS	[86]
Determination of Tl(I) and In(III) in tonic water by using a multivariate standard addition method	A screen-printed carbon nanofibers electrode modified with selenocystine and a screen-printed carbon electrode modified with a Bi film	PLS	[19]
Determination of the polyphenolic content of extra virgin olive oils	Twelve sensors: five of them based on lanthanide bisphthalocyanines, six based on polypyrrole and one unmodified carbon paste electrode.	PLS	[87]
Determination of bitterness index in olive oils	Six polypyrrole-based screen-printed electrodes	PLS	[65]
Quantification of total polyphenol content in olive oils	Polypyrrole modified screen-printed electrodes	PLS	[88]
Detection of antibiotic residues in bovine milk	Au, Ag, Pt, Pd, Ti	PCR, PLS, SVM	[89]
Determination of the antioxidant activity of camu camu and tumbo juices	Au, Pt, Ir, Rh, Ag, Cu, Ni, Co	PLS	[90]
